# Long-term survival with immune checkpoint inhibitors combined with bevacizumab in advanced clear cell renal cell carcinoma: A case report

**DOI:** 10.1097/MD.0000000000051079

**Published:** 2026-10-09

**Authors:** Jing Zhang, Chun-Qiao Chen, Hui Huang, Zhi-Chao Yao, Xi Qin, Fei-Fei Wei

**Affiliations:** a Department of Oncology, People’s Hospital of Guilin, Guilin, Guangxi Zhuang Autonomous Region, China; b Department of Pathology, People’s Hospital of Guilin, Guilin, Guangxi Zhuang Autonomous Region, China; c Department of Oncology, The First Affiliated Hospital of Guilin Medical University, Guilin, Guangxi Zhuang Autonomous Region, China; d Department of Tumor Radiotherapy, Nanxishan Hospital of Guangxi Zhuang Autonomous Region (The Second People’s Hospital of Guangxi Zhuang Autonomous Region), Guilin, Guangxi Zhuang Autonomous Region, China.

**Keywords:** bevacizumab, Case report, Clear cell renal cell carcinoma, Immune checkpoint inhibitors, Overall survival

## Abstract

**Rationale:**

Renal cell carcinoma is a highly heterogeneous urological malignancy, with clear cell renal cell carcinoma (ccRCC) as the most common pathological subtype. A considerable proportion of patients are diagnosed at advanced stages or experience postoperative recurrence and distant metastasis, leading to poor long-term prognosis. Although targeted therapy and immune checkpoint inhibitors (ICIs) have greatly improved the treatment landscape of advanced ccRCC, optimal sequential and combination treatment strategies for heavily pretreated metastatic patients remain to be further explored.

**Patient concerns:**

A 49-year-old male patient was diagnosed with stage IV ccRCC complicated with lung metastasis. After palliative nephrectomy and multiple lines of targeted and immunotherapeutic regimens, the patient still suffered from continuous tumor progression, with recurrent pulmonary metastatic lesions, newly occurring adrenal metastasis and brain metastasis, accompanied by clinical symptoms including cough, hemoptysis, and persistent dizziness.

**Diagnoses:**

The patient was definitively diagnosed with metastatic ccRCC (T1N1M1, Stage IV). Radiological examinations confirmed progressive pulmonary metastases, new right adrenal metastasis, and right frontal lobe brain metastasis after multiple lines of treatment.

**Interventions:**

The patient initially underwent palliative left nephrectomy and sequential sorafenib targeted therapy. After disease progression, intracranial intensity-modulated radiotherapy combined with sunitinib plus interleukin-2 immunotherapy was administered. Following subsequent disease progression, the patient received 4 cycles of camrelizumab combined with bevacizumab, followed by bevacizumab maintenance therapy. Since January 2024, the patient has been treated with tislelizumab plus bevacizumab combination regimen.

**Outcomes:**

After sequential multi-line individualized treatment, the patient achieved sustained stable disease. The overall survival of the patient has reached approximately 6 years up to the latest follow-up, with favorable long-term survival benefit and well-tolerated treatment safety.

**Lessons:**

For heavily pretreated metastatic ccRCC patients with prior targeted therapy but no previous ICI exposure, ICI combined with bevacizumab is a feasible and effective salvage treatment regimen, which can achieve durable survival benefits. Further prospective clinical studies and long-term follow-up are required to verify the clinical promotion value of this combination strategy.

## 1. Introduction

Renal cell carcinoma (RCC) is one of the common malignant tumors in the urinary system and a complex and heterogeneous disease. It arises from the malignant transformation of renal tubular epithelial cells, accounting for 2% to 3% of all adult malignancies.^[1]^ Clear cell renal cell carcinoma (ccRCC) is the predominant subtype, comprising 70%–75% of all RCC cases. Papillary renal cell carcinoma and chromophobe renal cell carcinoma account for 10% and 5% respectively, and 4–6% of renal cell carcinoma cannot be accurately classified.^[2]^ Approximately 20% to 30% of patients present with advanced disease at initial diagnosis. Surgery remains the standard treatment for localized RCC, yet approximately 20% of patients develop postoperative recurrence or distant metastasis after curative nephrectomy.^[3]^ Patients with advanced renal cancer often experience symptoms such as hematuria, back pain, and abdominal masses, with a low five-year survival rate, significantly impacting their quality of life and survival. With the continuous innovation of antitumor therapeutic agents, clinical management paradigms have shifted toward combination regimens, which yield superior tumor control and survival outcomes compared with single-agent targeted agents. Targeted therapy with immune checkpoint inhibitors (ICIs) and anti-vascular endothelial growth factor (anti-VEGF) antibody/tyrosine kinase inhibitors (TKIs) is gradually being applied in the treatment of advanced RCC.^[2]^ In this report, we conducted a retrospective analysis of a patient with advanced ccRCC, exploring the efficacy and safety of comprehensive treatment with multiple methods for RCC.

## 2. Case presentation

### 2.1. Chief complaints

A 49-year-old male patient was admitted with a 2-month history of recurrent painless gross hematuria.

### 2.2. History of present illness

Recurrent painless hematuria for 2 months.

### 2.3. History of past illness

No history of hypertension, diabetes, tuberculosis, hepatitis, or allergies.

### 2.4. Personal and family history

He denied occupational exposure to toxic or radioactive agents, and no familial history of hereditary malignancies was reported.

### 2.5. Physical examination

Temperature 36.6°C; heart rate: 60 beats per minute; respiratory rate: 20/min; blood pressure: 130/81 mm Hg. No abnormal rales were detected on bilateral lung auscultation. Regular heart rhythm. The abdomen was soft, with no focal tenderness or palpable mass. No tenderness in the liver and kidney areas, no edema in the lower limbs, and no neurological abnormalities were identified on physical examination. Karnofsky Performance Status (KPS) score was 90. Numeric rating scale (NRS) score was 0. According to the Khorana VTE risk factor assessment form, the tumor was categorized as moderate VTE risk (1 point).

### 2.6. Laboratory examinations

Urinalysis revealed 3 + occult blood and 3 + red blood cells (RBCs). Pathological examination of the left nephrectomy specimen revealed a tumor measuring 6 × 6 × 5 cm, with multiple nodules that involved the entire renal parenchyma and were adjacent to the renal pelvis. Microscopy showed nests of eosinophilic and clear tumor cells, some with enlarged nuclei and prominent nucleoli. Immunohistochemistry: VIM (+), CK7 (−), CA-IX (+), CD10 (focal weak +), RCC (focal +), CD117 (−), Ki-67 (proliferation index of 10–20%) (Fig. 1A).

**Figure 1.**
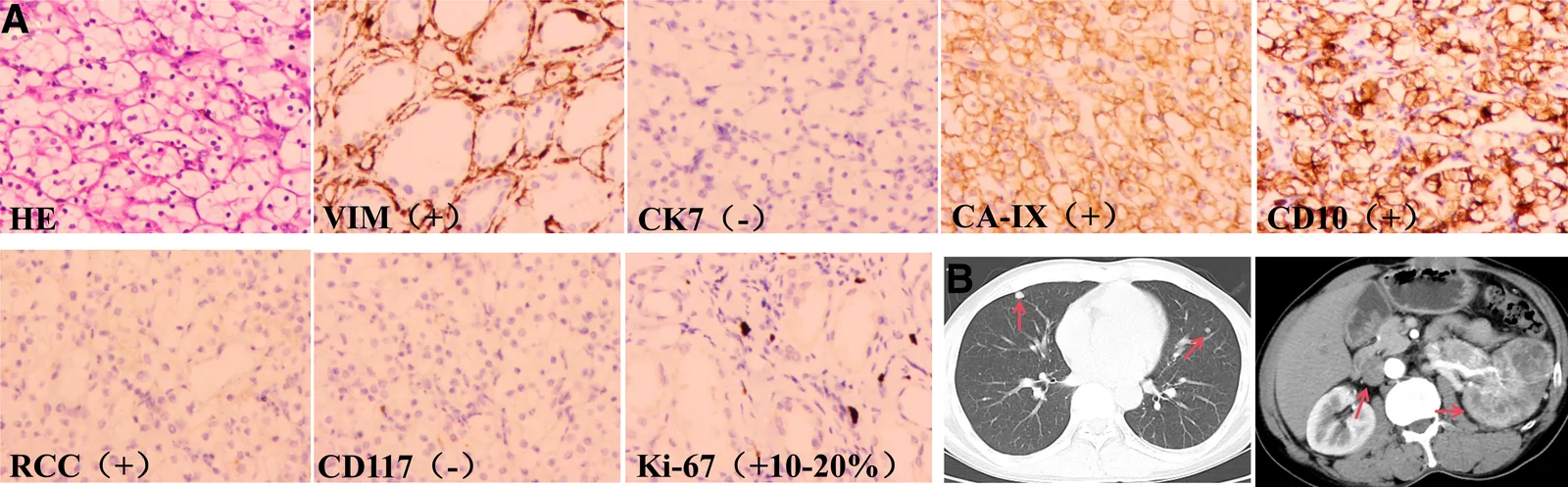
Pathological and radiological findings of the patient diagnosed with stage IV clear cell renal cell carcinoma (ccRCC) with pulmonary metastases (T1N1M1). (A) Histopathological and immunohistochemical staining of the primary renal tumor. Hematoxylin and eosin (HE) staining shows clear and eosinophilic tumor cells with enlarged nuclei; immunohistochemistry demonstrated positive expression of VIM, CA-IX, focal CD10 and RCC, negative CK7 and CD117, with a Ki-67 proliferation index of 10–20%. (B) Baseline computed tomography (CT) images at initial diagnosis. Left panel: Chest CT showing multiple bilateral pulmonary metastatic nodules; Right panel: Urinary tract CT revealing a left renal mass complicated by renal vein tumor thrombus, at the position indicated by the arrow.

### 2.7. Imaging examinations

The chest X-ray showed multiple metastatic nodules in both lungs. Computed tomography (CT) of the urinary tract revealed left renal carcinoma with a renal vein tumor thrombus (Fig. 1B).

## 3. Final diagnosis

Left ccRCC with lung metastases (T1N1M1, Stage IV).

## 4. Treatment

In April 2018, the patient underwent palliative resection of the left renal tumor. In June 2019, sorafenib was administered for targeted therapy to treat lung metastases. Between December 2022 and January 2023, the patient received static field intensity-modulated radiotherapy (SF-IMRT, 6 MV X-ray) targeting the right frontal lobe metastatic lesion. A total dose of 6000 cGy was prescribed to cover 95% of the planning gross tumor volume (PGTV), delivered across 20 fractions over 29 days. Concurrently, combination therapy with sunitinib (50 mg qd) and IL-2 (2 million IU on days 1–5) was implemented.

By April 2023, imaging examinations revealed that the number and size of nodules in both lungs had increased compared to before, considered as progression of bilateral pulmonary metastases. Subsequently, the patient received 4 cycles of camrelizumab (200 mg on day 2) plus bevacizumab (600 mg on day 1) every 3 weeks (q3w) from April to July 2023, followed by single-agent bevacizumab (600 mg q3w) maintenance therapy until October 2023.

In January 2024, progressive enlargement of bilateral pulmonary metastases and new lesions in the left frontal lobe necessitated a treatment switch to tislelizumab (200 mg) plus bevacizumab (600 mg), which has been maintained up to the last follow-up.

## 5. Outcome and follow-up

Approximately one year after surgery, follow-up chest CT revealed increased bilateral pulmonary nodules. A new lymph node shadow was observed below the trachea. The patient was prescribed sorafenib for targeted therapy, and the treatment was evaluated as stable disease (SD).

In November 2022, the patient experienced a paroxysmal dry cough, followed by hemoptysis and dizziness. CT in December 2022 showed a new right adrenal lesion and significant enlargement of bilateral pulmonary nodules. The nodules in the right bronchus, right pulmonary hilum, and mediastinal lymph nodes were enlarged and fused, with significant enlargement compared to before. A brain MRI revealed a metastatic tumor in the right frontal lobe, indicating disease progression (PFS: 41 months). After receiving sunitinib treatment, the patient’s cough and hemoptysis improved. Following IMRT for the brain metastasis combined with IL-2 and sunitinib targeted therapy, the patient’s condition stabilized.

By April 2023, follow-up chest CT imaging showed further progression of bilateral pulmonary nodules and enlargement of the right adrenal metastasis. Treatment response was assessed as progressive disease (PD), with a corresponding PFS of 4 months. The treatment was switched to bevacizumab (600 mg) plus camrelizumab (200 mg). Subsequent imaging showed shrinkage of the right adrenal metastasis, bilateral pulmonary lesions, and hilar/mediastinal lymph nodes.

During the treatment with camrelizumab combined with bevacizumab (April to July 2023) and bevacizumab maintenance therapy (until October 2023), the patient was closely monitored for adverse events. No obvious hypertension, proteinuria, gastrointestinal reactions (such as nausea, vomiting, diarrhea), or hematological toxicity (such as leukopenia, thrombocytopenia) were observed. Grade 1 fatigue was the only treatment-related adverse event, which resolved completely with rest without the need for a dose hold or dose reduction.

In January 2024, chest CT and head MRI showed that the metastases had increased in size compared to before, and new lesions were visible in the left frontal and temporal lobes, considering tumor progression (PFS: 9 months). The patient was transitioned to tislelizumab (200 mg) plus bevacizumab (600 mg). posttreatment efficacy via brain MRI, chest, and abdominal CT was evaluated as a partial response (PR). When the treatment was switched to tislelizumab combined with bevacizumab in March 2024, the patient experienced recurrent limb myoclonus. Comprehensive evaluations, including electroencephalography and brain MRI, confirmed a diagnosis of tumor-induced secondary epilepsy. The treatment was adjusted by adding sodium valproate (500 mg twice a day) for anti-epilepsy treatment. During the treatment, the doses of tislelizumab and bevacizumab were maintained without treatment interruption. After 2 weeks of treatment with sodium valproate, the limb twitching symptoms disappeared, and no recurrence was observed during the subsequent follow-up.

Subsequent brain MRI in December 2024 demonstrated marked enlargement of the metastatic lesion in the left parietal lobe. The patient has been treated with a combination of tislelizumab and bevacizumab until now. Throughout the course of the combination therapy, no other severe treatment-related adverse events (grade 3 or above) were observed, and the patient’s KPS score remained at 80–90, indicating good tolerability of the regimen. Treatment efficacy was evaluated as SD, with an overall survival (OS) of approximately 6 years to date (Fig. 2, Table 1).

**Table 1:** The treatment regimen.

Date	Treatment Process	Outcomes and Follow-up
2018.04	Palliative resection of the left renal tumor. Followed by sorafenib treatment.	/
2019.06	Follow-up chest CT revealed increased bilateral pulmonary nodules. A new lymph node shadow was observed below the trachea. Initiated sorafenib for pulmonary metastases.	Treatment efficacy was evaluated as stable disease (SD).
2022.11	Experienced a paroxysmal dry cough, followed by hemoptysis and dizziness.CT revealed a new right adrenal lesion, significant enlargement of bilateral pulmonary nodules. The nodules in the right bronchus, right pulmonary hilum, and mediastinal lymph nodes were enlarged and fused. Brain MRI showed a right frontal lobe metastasis.	Disease progression, PFS 41 months.
2022.12–2023.01	Intensity modulated radiotherapy (6MV X-ray SF-IMRT Dt 95% PGTV: 6000cGy/20f/29d) for brain metastases, combined with sunitinib (50 mg qd) and IL-2 (2 million IU on days 1–5).	/
2023.04	Follow-up chest CT imaging showed further progression of bilateral pulmonary nodules and enlargement of the right adrenal metastasis.	Treatment efficacy was evaluated as progressive disease (PD), PFS 4 months.
2023.04–2023.10	4 cycles of Bevacizumab (600 mg on day 1) combined with Camrelizumab (200 mg on day 2) were administered from April to July 2023, followed by maintenance therapy with Bevacizumab (600 mg) until October 2023.	/
2024.01	Chest CT and head MRI showed that the metastases had increased in size compared to before, and new lesions were visible in the left frontal and temporal lobes.	Disease progression, PFS 9 months.
2024.01–2024.10	8 cycles of Tislelizumab 200 mg + Bevacizumab 600 mg was administered.	Treatment efficacy was evaluated as partial response (PR).
2024.12	head MRI showed a significant increase in the size of the left parietal lobe lesion compared to before.	/
2024.12–2025.04	4 cycles of treatment with Tislelizumab 200 mg + Bevacizumab 600 mg was administered.	Treatment efficacy was evaluated as SD with an overall survival (OS) of approximately 6 years to date.

**Figure 2.**
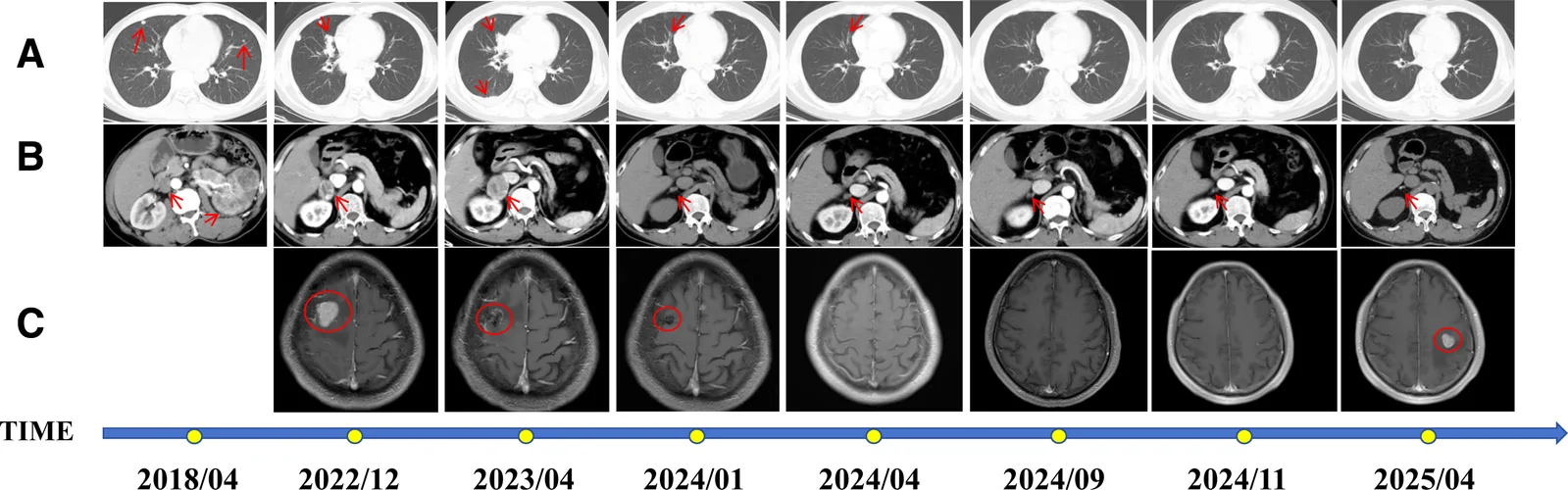
Serial radiological follow-up images and longitudinal treatment timeline of the patient with advanced ccRCC. The figure contains sequential chest computed tomography (CT), abdominal CT, and brain magnetic resonance imaging (MRI) scans collected at multiple key time points during treatment. Red arrows indicate (A) pulmonary metastatic lesions and (B) right adrenal metastatic masses, while red circles mark (C) intracranial metastatic tumors in the frontal/temporal/parietal lobes. The timeline below the images records the full treatment sequence and corresponding tumor response assessment from April 2018 to February 2025. •2018.04: Baseline imaging after left nephrectomy; •2022.12: Disease progression with new adrenal and brain metastases; •2023.04: Progressive disease after sunitinib + IL-2 and radiotherapy; •2024.01: Tumor progression following camrelizumab + bevacizumab maintenance; •2024.04–2025.04: Long-term disease control with tislelizumab combined with bevacizumab, achieving partial response (PR) then stable disease (SD).

## 6. Discussion

This case illustrates the efficacy and safety of multimodal therapy for advanced ccRCC. After progression on first-line surgery and sorafenib, switching to another TKI combined with radiotherapy and IL-2 provided benefit. Subsequent progression led to ICI (camrelizumab then tislelizumab) combined with bevacizumab, with ongoing bevacizumab maintenance, achieving an OS of 6 years with manageable toxicity.

Over the past few decades, the treatment methods for advanced RCC have undergone significant changes. Initially, interleukins and interferons were the primary treatments, but they did not significantly improve survival rates and were accompanied by severe side effects.^[3]^ In recent years, a combination therapy strategy centered on various targeted drugs has been actively explored in the treatment of metastatic RCC. This approach has shown some improvement in disease control rate (DCR), objective response rate (ORR), and patient PFS and OS, but the overall efficacy remains unsatisfactory. Currently, preclinical and clinical studies suggest that the combination of targeted drugs affecting different pathways of tumor growth and signal transduction could enhance treatment outcomes.^[2]^ RCC targeted therapies are categorized into TKIs, anti-VEGF monoclonal antibodies, ICIs, and mammalian target of rapamycin (mTOR) inhibitors.^[4]^ Recently, ICIs-based combinations have revolutionized the treatment landscape for advanced RCC.^[3]^

This case reports a patient with advanced ccRCC with lung and brain metastasis. Approximately 10% of RCC patients develop brain metastases, with a median overall survival (OS) of <3 months.^[5]^ Advanced RCC is a highly vascularized tumor, and anti-angiogenic agents such as TKIs and mTOR inhibitors are currently the primary treatment options.^[4]^ Sorafenib, sunitinib, and Cabozantinib are standard first-line options for ccRCC. In this case, sorafenib was administered as post-nephrectomy systemic therapy, followed by sunitinib upon progression. Due to the blood-brain barrier, the ORR of targeted therapy for RCC brain metastases is only 4%~9%.^[5]^ For brain metastases, stereotactic radiotherapy has shown effectiveness and safety for oligometastasis.^[6]^ Klausner et al^[7]^ reported that the DCR of intracranial metastases is primarily influenced by the dose of radiotherapy and the use of drugs. It is higher when the dose of stereotactic body radiotherapy exceeds 17 Gy/1 fraction and TKIs are used concurrently. Based on this, the patient underwent IMRT for brain metastases. The combination of targeted therapy, radiotherapy, and IL-2 has shown some efficacy in improving disease control and survival, with the patient PFS reaching approximately 41 months. Studies have shown that the objective response rate of interferon-α and IL-2 immunotherapy for advanced RCC is merely 5% to 20%.^[2]^ After the disease progressed again, we explored more aggressive treatment options.

Numerous studies have shown that programmed death ligand-1 (PD-L1) is highly expressed in ccRCC, and there is a significant infiltration of immune cells, suggesting that immunotherapy with ICIs may play a crucial role in the treatment of RCC.^[8]^ In pathological conditions, tumor cells can constitutively express PD-L1/PD-L2, which binds to the PD-1 receptor on T lymphocytes. This binding triggers intracellular tyrosine phosphorylation of PD-1 cytoplasmic domains, further inducing dephosphorylation of key T-cell receptor signaling molecules. Consequently, downstream T-cell activation signals are blocked, suppressing T-cell proliferation and cytotoxic function and enabling tumors to evade immune surveillance.^[1]^ tislelizumab, a novel PD-1 inhibitor, can competitively bind to PD-1 using multiple complementary sequences from the variable heavy-variable light domain, thereby restoring the transmission of downstream signals in T cells and enhancing the activity of immune T cells.^[9]^ A Phase I clinical trial showed patients with advanced RCC treated with tislelizumab monotherapy achieved an ORR of 31.3%, and most adverse reactions were mild.^[10]^ Furthermore, Wang et al^[11]^ have reported that Axitinib combined with tislelizumab demonstrated superior ORR (59.1%) and DCR (81.8%) compared to Axitinib alone (40.7%, 66.7%). This indicates that combining Axitinib with tislelizumab can significantly enhance anticancer efficacy. Additionally, it is worth noting that multiple Phase III trials have confirmed favorable clinical outcomes for diverse ICIs-based combination regimens. Nevertheless, high-level Phase III evidence supporting frontline single-agent ICIs for metastatic or unresectable ccRCC remains absent; accordingly, monotherapy with ICIs is not recommended for patients with advanced ccRCC.^[12]^ In this case, at the time of deciding on the second-line treatment (April 2023), the patient had experienced disease progression after 2 lines of TKI therapy (sorafenib, sunitinib) and stereotactic IMRT for brain metastases, indicating that targeted therapy alone has certain limitations in advanced RCC. Based on disease progression, we hoped to achieve a better therapeutic effect by using a combination therapy with potential synergistic effects to control the progressive disease. Studies indicate that in a phase II clinical trial of sorafenib combined with bevacizumab as first-line targeted therapy for metastatic RCC, tumors showed varying degrees of shrinkage, with the proportion of patients achieving stable disease at 6 months reaching 59% and a median PFS of 9.2 months.^[13]^ Another randomized controlled phase II clinical trial (BEST study) evaluating sorafenib in combination with bevacizumab for metastatic RCC reported an ORR of 30% and a median PFS of 11.3 months.^[14]^ Currently, few studies have been conducted on combining ICIs with anti-angiogenic agents for advanced RCC. The clinical prospects of VEGFR-TKI monotherapy and the durable responses possible with ICIs therapy have driven the investigation into combined VEGFR-TKI/ICIs strategies. The phase II NeoAvAx trial (NCT03341845)^[15]^ evaluated neoadjuvant avelumab plus axitinib in 40 patients with high-risk localized clear cell renal cell carcinoma. After 12 weeks of treatment, the median tumor reduction was 20%, with 30% (12/40) of patients achieving a partial response. At a median follow-up of 23.5 months, 67.5% of patients remained disease-free, including 83% of those who had a partial response. The regimen demonstrated promising efficacy and feasibility, although longer follow-up is needed to assess survival impact. In addition, a phase II study (NCT04118855)^[16]^ neoadjuvant toripalimab plus axitinib in 20 patients with locally advanced clear cell RCC resulted in a median tumor reduction of 26.7% and an ORR of 45%, including four pathologic complete responses. At 21.3 months median follow-up, 20% of patients experienced recurrence. Treatment was well-tolerated without grade 4/5 AEs. Responders showed higher baseline immune cell infiltration. In another phase II trial (NCT05172440)^[17]^ with tislelizumab plus axitinib, an interim analysis of nine high-risk nonmetastatic RCC patients showed an ORR of 55.5% and median tumor reduction of 26.3%. The combination facilitated surgical downstaging in 3 patients and had no grade 4/5 adverse events (AEs) or surgery-related complications. Collectively, these novel VEGFR-TKI and ICIs combinations demonstrate considerable potential for achieving tumor regression. In this case, the combination of tislelizumab and bevacizumab achieved sustained tumor control, achieving an OS of approximately 6 years, and the patient’s condition remains relatively stable. This indicates that the combination therapy of ICIs and anti-angiogenic agents achieves a higher ORR and PFS in advanced RCC compared to targeted therapy alone.

Analysis of the underlying mechanisms suggests that anti-angiogenic agents may restore T-cell function by reversing VEGF-mediated suppression of antigen presentation in dendritic cells. This promotes T-cell infiltration into tumor tissue while reducing populations of myeloid-derived suppressor cells and immunosuppressive regulatory T cells. Consequently, the immunosuppressive microenvironment transforms into an immune-supportive milieu. Meanwhile, ICIs enhance the antitumor capacity of T cells. This complementary action establishes the anti-angiogenic/ICI combination as a synergistic “golden combination” that amplifies the body’s antitumor immune response. The synergistic antitumor activity of anti-angiogenic agents combined with ICIs can be explained by complementary immunomodulatory mechanisms within the tumor microenvironment. Furthermore, compared to conventional PD-1 inhibitors, tislelizumab was engineered with Fc domain modification during its development. This Fc-domain modification abolishes binding to macrophage Fc receptors, which mitigates T-cell depletion driven by antibody-dependent cellular phagocytosis (ADCP),^[9]^ which can also reduce adverse reactions and delay the onset of drug resistance. The patient had not received ICI treatment before, so the use of ICI-based combination therapy could provide a new treatment option for the patient.

In managing complex clinical scenarios, treatment strategies should be tailored to individual patient profiles. Following failure of TKI monotherapy or targeted-immunotherapy combinations, switching to alternative TKIs or ICIs, or opting for combined targeted-immunotherapy may be considered for subsequent treatment. In this case, the sustained efficacy of combining ICIs with bevacizumab likely contributed to the observed long-term survival. Observations from this single-case report cannot be generalized; large-scale prospective clinical trials are mandatory to validate the efficacy and safety of this combination regimen prior to routine clinical use.

## 7. Conclusion

For patients with metastatic or unresectable ccRCC who received sequential lines of targeted therapy without prior ICI exposure, the combination of ICIs and bevacizumab may confer clinical benefits, including prolonged PFS and OS. Prospective clinical trials are needed to validate the efficacy of this ICI plus bevacizumab regimen, and long-term follow-up studies are required to characterize its durable survival benefits prior to widespread clinical implementation.

## Author contributions

**Writing—original draft:** Jing Zhang.

**Writing—review & editing:** Jing Zhang, Fei-Fei Wei.

**Data curation:** Chun-Qiao Chen.

**Investigation:** Hui Huang.

**Validation:** Hui Huang, Xi Qin.

**Formal analysis:** Zhi-Chao Yao.

**Funding acquisition:** Fei-Fei Wei.

## References

[1] Nezami BG MacLennan GT. Clear cell renal cell carcinoma: a comprehensive review of its histopathology, genetics, and differential diagnosis. Int J Surg Pathol. 2025;33:265–80. 10.1177/10668969241256111 39051572

[2] Chen YW Wang L Panian J. Treatment landscape of renal cell carcinoma. Curr Treat Options Oncol. 2023;24:1889–916. 10.1007/s11864-023-01161-5 PMC10781877 38153686

[3] Gulati S Labaki C Karachaliou GS Choueiri TK Zhang T. First-line treatments for metastatic clear cell renal cell carcinoma: an ever-enlarging landscape. Oncologist. 2022;27:125–34. 10.1093/oncolo/oyab056 PMC8895741 35641205

[4] Park JA Espinosa-Cotton M Guo HF Monette S Cheung NV. Targeting tumor vasculature to improve antitumor activity of T cells armed ex vivo with T cell engaging bispecific antibody. J ImmunoTher Cancer. 2023;11:e006680. 10.1136/jitc-2023-006680 PMC10069597 36990507

[5] Sternberg CN Calabrò F Bracarda S. Safety and efficacy of sunitinib in patients from Italy with metastatic renal cell carcinoma: final results from an expanded-access trial. Oncology (Huntingt). 2015;88:273–80. 10.1159/000369256 25592399

[6] Liu Y Long W Zhang Z. Metastasis-directed stereotactic body radiotherapy for oligometastatic renal cell carcinoma: extent of tumor burden eradicated by radiotherapy. World J Urol. 2021;39:4183–90. 10.1007/s00345-021-03742-1 PMC8571216 34043023

[7] Klausner G Troussier I Biau J. Stereotactic radiation therapy for renal cell carcinoma brain metastases in the tyrosine kinase inhibitors era: outcomes of 120 patients. Clin Genitourin Cancer. 2019;17:191–200. 10.1016/j.clgc.2019.02.007 30926219

[8] Zhu Z Jin Y Zhou J. PD1/PD-L1 blockade in clear cell renal cell carcinoma: mechanistic insights, clinical efficacy, and future perspectives. Mol Cancer. 2024;23:146. 10.1186/s12943-024-02059-y PMC11251344 39014460

[9] Yi M Zheng X Niu M Zhu S Ge H Wu K. Combination strategies with PD-1/PD-L1 blockade: current advances and future directions. Mol Cancer. 2022;21:28. 10.1186/s12943-021-01489-2 PMC8780712 35062949

[10] Desai J Deva S Lee JS. Phase IA/IB study of single-agent tislelizumab, an investigational anti-PD-1 antibody, in solid tumors. J ImmunoTher Cancer. 2020;8:e000453. 10.1136/jitc-2019-000453 PMC7295442 32540858

[11] Wang Z Chen J Li Q Li N Yu J Liu F. The effects of axitinib plus tislelizumab in the treatment of advanced renal cell carcinoma. J Oncol. 2022;2022:2700166. 10.1155/2022/2700166 PMC8970885 35368892

[12] Chinese Expert Consensus on The Systemic Treatment of Advanced Clear Cell Renal Cell Carcinoma Workgroup; Chinese Society of Clinical Oncology, Experts Committee on Renal Carcinoma; China Anti-Cancer Association, The Society of Genitourinary Tract Cancer. Chinese expert consensus on the systemic treatment of advanced clear cell renal cell carcinoma (2024 edition). Zhonghua Zhong Liu Za Zhi. 2024;46:844–54. Chinese 10.3760/cma.j.cn112152-20240322-00117 39293987

[13] Flaherty KT Manola JB Pins M. BEST: a randomized phase II study of vascular endothelial growth factor, RAF kinase, and mammalian target of rapamycin combination targeted therapy with bevacizumab, sorafenib, and temsirolimus in advanced renal cell carcinoma--a trial of the ECOG-ACRIN Cancer Research Group (E2804). J Clin Oncol. 2015;33:2384–91. 10.1200/JCO.2015.60.9727 PMC4500832 26077237

[14] McDermott DF Manola J Pins M. The BEST trial (E2804): a randomized phase II study of VEGF, RAF kinase, and mTOR combination targeted therapy (CTT) with bevacizumab (bev), sorafenib (sor), and temsirolimus (tem) in advanced renal cell carcinoma (RCC). J Clin Oncol. 2013;31:345–345. 10.1200/JCO.2015.60.9727 PMC4500832 26077237

[15] Bex A Abu-Ghanem Y Van Thienen JV. Efficacy, safety, and biomarker analysis of neoadjuvant avelumab/axitinib in patients (pts) with localized renal cell carcinoma (RCC) who are at high risk of relapse after nephrectomy (NeoAvAx). J Clin Oncol. 2022;40(Suppl 6):289–289.

[16] Huang J Wang Y Xu F. Neoadjuvant toripalimab combined with axitinib in patients with locally advanced clear cell renal cell carcinoma: a single-arm, phase II trial. J ImmunoTher Cancer. 2024;12:e008475. 10.1136/jitc-2023-008475 PMC11168135 38862251

[17] Zhang S Ji C Liu G Guo H. A phase II study of neoadjuvant tislelizumab and axitinib in patients with locally advanced non-metastatic clear cell renal cell carcinoma (accRCC). J Clin Oncol. 2024;42(Suppl 4):420–420.

